# Precancerous Cervical Lesion Among Adult Women With Human Immune Deficiency Virus on Anti Retroviral Therapy At Saint Peter Specialized Hospital, Ethiopia: A Hospital-Based Cross-Sectional Study

**DOI:** 10.3389/fonc.2022.910915

**Published:** 2022-07-22

**Authors:** Wondimu Zelalem, Fitsum Weldegebreal, Behailu Hawulte Ayele, Alemayehu Deressa, Adera Debella, Addis Eyeberu, Fila Ahmed Hassen, Yadeta Dessie

**Affiliations:** ^1^ School of Public Health, Saint Peter Specialized Hospital, Addis Ababa, Ethiopia; ^2^ School of Medical Laboratory Sciences, College of Health and Medical Sciences, Haramaya University, Harar, Ethiopia; ^3^ Department of Public Health and Health Policy, School of Public Health, Haramaya University, Harar, Ethiopia; ^4^ School of Nursing and Midwifery, College of Health and Medical Sciences, Haramaya University, Harar, Ethiopia

**Keywords:** precancerous cervical lesion, HIV, ART, prevalence, Ethiopia

## Abstract

**Background:**

Cervical cancer is the fourth most frequent cancer in women representing 6.6% of all female cancers occurring in low and middle-income countries, where resources for cancer prevention programs are often scarce. So this study aimed to assess the prevalence of precancerous cervical lesion and associated factors among adult women with human immune deficiency virus (HIV) on Anti Retroviral Therapy (ART) at Saint Peter Specialized Hospital, Addis Ababa, Ethiopia.

**Methods:**

An institution-based cross-sectional study was conducted from November 06 to July 20, 2020 among 267 adult women with HIV on Anti Retroviral Therapy At Saint Peter Specialized Hospital, Ethiopia. Data were collected using face-to-face interview, patient chart review, and the examination of the squamo-columnar junction by the visual inspection with the acetic acid method. The collected data were entered into Epi-data version 3.1 and exported to Statistical Package for the Social Sciences (SPSS) version 24.0 for analysis. Bivariate and multivariable binary logistic regression analysis were used to identify factors associated with the precancerous cervical lesion. Statistical significance was considered at a P-valve less than 0.05.

**Result:**

A total of 267 women who were on ART were included in the study and the prevalence of precancerous cervical lesion was 7.5% with 95% CI =4.10%-10.50%. Modern family planning (AOR = 4.14, 95% CI = 1.23-13.87), history of sexual transmission infection (STI) (AOR=5.39, 95% CI= 1.56-18.70) and viral load (AOR=20.85, 95% CI = 6.19-70.25) had significant association with precancerous cervical lesion

**Conclusion:**

In this study, the prevalence of precancerous cervical lesion is relatively low compared to studies in low and middle-income countries. Modern family planning, history of sexual transmitted infection, and viral load had a significant association with a precancerous cervical lesion. Hence, encouraging modern family planning, and routine screening of women for pre-cancerous cervical lesions for those with high viral load have enormous contributions to decreasing cervical cancer disease among Women with Human Immune Deficiency Virus through Anti Retroviral Therapy.

## Introduction

Despite the fact that cervical cancer is preventable through vaccination, screening, and treatment, approximately 570 000 cases of the disease and 311 000 deaths occurred worldwide in 2018. However, cervical cancer is the fourth leading cause of cancer deaths in women globally, and nearly 90% of cervical cancer deaths occurred in the developing world. Sub-Saharan Africa contains about 22% of global cervical cancer cases (1–3). The number of deaths is expected to rise by 67% by 2030, reaching 443,000 (4) In Ethiopia, an estimated 7000 new cases and 5000 deaths from cervical cancer occur each year (5).

The disproportionately high prevalence of cervical cancer in Sub-Saharan Africa, parts of Latin America and the Caribbean, and other medically underserved populations are primarily due to a lack of screening (3). Human papillomavirus (HPV), a sexually transmitted infection, is associated with a higher prevalence of HIV infection, especially in Sub-Saharan Africa (6–9); and in Ethiopia such to human papillomavirus (HPV), cultural factors, poverty, co-infection, and lack of awareness are common risk factors of cervical cancer (10). Furthermore, there is no standard policy or protocol for cervical cancer screening in Ethiopia; rather, it is patchy or inconsistent, and Ethiopian women typically present for cancer care at a late stage in the disease, when treatment is likely ineffective (5).

Screening high-risk and vulnerable populations for asymptomatic precancerous cervical lesions reduce the burden of cervical cancer-related morbidities and mortalities significantly (11) Ethiopia is currently implementing a Precancerous cervical lesion (PCCL) screening program in order to meet the 2020 target of screening at least 80% of 30-49-year-old women (12) and takes the cervical cancer crisis seriously, particularly because 99% of HIV+ women received a VIA test (13). Despite various prevention and control strategies, cervical cancer is still on the rise in Ethiopia. According to the 2016 Ethiopian demographic health survey (EDHS), the prevalence of HIV/AIDS among women aged 15 to 49 years was 1.2% (14). In Ethiopia, 534,000 women aged 15 and above are infected with HIV. These women are among the most vulnerable to cervical cancer because their risk of pre-cancerous lesions is ten times higher and they are more likely to develop invasive cervical cancer than uninfected women (9, 15).

According to WHO guidelines, every sexually active woman aged 30–49 years should have cervical cancer screening at least every 5 years. However, sexually active and HIV-positive women of any age should be screened every three years (1) Ethiopia adopted the WHO recommendation in 2015, recommending that HIV-positive women begin screening at HIV diagnosis, regardless of age, and re-screen every 5 years (15). Ethiopia’s government has placed a greater emphasis on programs aimed at detecting cervical cancer at an early stage. To combat cervical cancer, various stakeholders such as academia, professionals, the media, and development partners have made several advocacy efforts (15).

Despite the government’s commitment and the interventions of various stakeholders, the magnitude and major risk factors of Precancerous Cervical Lesions remain unabated. Moreover, to the knowledge of the investigator concerned, there is a paucity of research regarding predictors of Precancerous Cervical Lesions among adult women with HIV on Anti Retroviral Therapy at the study area level to help those working on the problem take evidence-based coordinated measures. As the result, the purpose of this study is to determine the magnitude and factors associated with precancerous cervical lesions among HIV-positive women who are on ART in St. Peter Specialized Hospital.

## Methods and Materials

### Study Design, Setting, and Period

An institutional-based cross-sectional study was conducted in St. Peter Specialized Hospital, Addis Ababa, the Capital City of Ethiopia from November 06 to July 20, 2020. St. Peter Specialized Hospital is one of the largest public hospital found in Addis Ababa city. The hospital was built in the early 1948G.C and currently, it has around 280 inpatient beds. The hospital is currently providing ART services for about 2300 HIV-positive clients by two medical doctors and 6 nurses. The hospital has started cervical cancer screening services since 2015 for all reproductive age women and has recently offered VIA cervical cancer screening and cryotherapy services.

### Population and Eligibility Criteria

All HIV Positive women at age of 18 up to 65 years, who were on ART follow-up and didn’t get cervical cancer screening services in the last five years were the study population. Those who were diagnosed with cervical cancer before the study period were excluded from the study.

### Sample Size and Sampling Procedures

The sample size was calculated using single population proportion formula with the assumptions of: Zα/2 = 1.96, 95% confidence level, 5% margin of error, and 20.2% proportion of precancerous cervical lesion among HIV positive women (taken from a study conducted in the Tigray Region, Northern Ethiopia (16). Since our study population was less than 10,000 (1540), finite population correction formula was used and the adjusted sample size was 242. By considering a 10% non-response rate, the final sample size was 267.

A systematic random sampling technique was used to select the study participants. Both primary and secondary data were collected through face-to-face interview, patient chart review, and visual inspection method.

#### Data Collection Tools, Procedures, and Quality Control

A structured questionnaire developed by reviewing other similar studies conducted before (8, 16, 17) was used to data related to socio-demographic and behavioral related factors. Wealth index tools were used in Ethiopia’s demographic health survey (14). A clinically related variable was collected from a patient chart review. Following the face-to-face interview study participants were tested for cervical cancer using visual inspection with acetic acid (VIA).

Trained nurses working in the precancerous cervical lesion screening and treatment centers of the Saint Peters hospitals were undertake the screening procedure and interviewed those patients after screening patients then review the patient chart.

#### Visual Inspection Methods

An un-lubricated bivalve speculum was inserted into the vagina and the cervix was visualized using a halogen focus lamp to identify the Squamo-Columnar Junction (SCJ). After cleaning away any excess mucus using a cotton swab, a five percent acetic acid solution was applied to the cervix for visual inspection with acetic acid. A finding was visible one minute after application. Precancerous lesions were defined as being dense aceto-white lesions with well-defined margins observed within the vicinity of the transformation zone originating from the SCJ, or if the whole cervix or cervical growth turned white (18). A suspicion of invasive cervical cancer was defined as any cervical ulcer or growth being observed. Results of VIA were classified as negative, positive, or suspicious for Invasive Cervical Cancer (ICC) according to the International Agency for Research on Cancer (IARC) training manual. Whenever there was uncertainty about the screening result, the nurses consulted a trained gynecologist, and he/she would confirm the diagnosis (19).

The questionnaire was initially prepared in English and then translated into the local language (Amharic language) by language experts then translated it back into English to ensure consistency. The data collectors and field supervisors received training on the data collection tool and procedures. Before the actual study, the pretest was conducted among 10% of study participants in similar settings. The investigators and experienced field research supervisors provided regular supervision.

### Operational Definition and Measurement

#### Precancerous Cervical Lesions

Defined as being dense aceto-white lesions with well-defined margins observed within the vicinity of the transformation zone originating from the SCJ, or if the whole cervix or cervical growth turned white during VIA. It was classified as negative, positive, or suspicious for Invasive Cervical Cancer (ICC) according to the International Agency for Research on Cancer (IARC) training manual (18).

#### Suspicious of Cervical Cancer

Any abnormal mass around the cervical and ulcer mass with an unusual sign and symptom, or in the 12 months prior (20).

#### Extensive/Large Lesion

If the lesion extends beyond the cryoprobe being used, or extends more than 2 mm into the cervical canal or the vaginal wall (21).

#### Alcohol Use

A study participant who drinks or has a history of drinking at least two bottles of beer per day or at least ten bottles of beer per week was considered an alcohol user (6).

### Cigarette Smoking

#### Current Smoker

A study participant who has smoked at least 100 cigarettes in her lifetime and who currently smokes cigarettes (22).

#### Former Smoker

A study participant who has smoked at least 100 cigarettes in her life but who had quit smoking at the time of interview (22).

Non-smoker: A study participant who has never smoked cigarettes, or who has smoked less than 100 cigarettes in her lifetime (22).

### Data Processing and Analysis

The collected data were checked for completeness, cleaned, coded, and entered into EpiData version 3.1. Then, the data were exported to SPSS version 24 for analysis. Descriptive and summary statistics were done and the information was presented using tables.

A binary logistic regression model was fitted to check for the association between independent variables and the outcome variable. The assumptions of binary logistic regression were checked. The goodness of fit was checked by Hosmer-Lemeshow statistic and omnibus tests. The assumptions were fulfilled and the model was fitted for the data. All variables with p < 0.25 in the bivariate analysis were included in the final multivariable analysis to identify the true predictors of precancerous cervical lesions among adult women with HIV on Anti Retroviral Therapy. Multi-collinearity test was carried out to check the presence of correlation between independent variables by using the standard error and co-linearity statistics but there was no multi-collinearity found among the variables. The direction and strength of statistical association were measured by odds ratio (OR) along with the 95% confidence interval (CI). P-value < 0.05 was considered to declare statistical significance.

## Results

### Socio-Demographic Characteristics

A total of 267 women who were on ART were included with a response rate of 100%. The mean age of study participants was 39.7 (SD ±8.08) years old, and 125 (46.8%) were in the age category of 31-40 years. Two hundred fifty-seven (96.3%) and 226 (84.6%) of study participants were urban residents and Orthodox followers, respectively. Eighty (30.0%) were unable to read and write and 13 (4.9%) had college or above education status. Housewife 106 (39.7%) and daily laborer 74 (27.7%) were more frequently mentioned occupations of study participants. The study participants’ mean ages at menarche, first sexual intercourse, and first marriage were 14.4 (1.08%) years, 19.4 (1.82%) years, and 22.5 (3.23%) years, respectively. There were 237 people who had two or more sexual partners at some point in their lives (88.9%). In terms of pregnancy history, the average age at first pregnancy was 23.7 (3.22%) (**Table 1**).

**Table 1:** Sociodemographic characteristics of study participants in Saint Peter Specialized Hospital, ART clinic, Ethiopia, 2020 (n=267).

Variables	Categories	Frequency	Percentage (%)	
Age in years	≤40	163	61.0	
	>40	104	39.0	
Residence	Rural	10	3.7	
	Urban	257	96.3	
Religion	Orthodox	226	84.6	
	Muslim	26	9.7	
	Protestant	15	5.6	
Ethnicity	Amhara	116	43.4	
	Oromo	81	30.3	
	Tigray	30	11.2	
	Gurage	33	12.4	
	Others*	7	2.6	
Educational status	Unable to read and write	80	30.0	
	Primary school	105	39.3	
	Secondary school	69	25.8	
	College and above	13	4.9	
Marital status	Single	51	19.1	
	Married	81	30.3	
	Separated	21	7.9	
	Widowed	43	16.1	
	Divorced	71	26.6	
Occupation	Housewife	106	39.7	
	Merchant	27	10.1	
	Gov’t employee	23	8.6	
	Private employee	29	10.9	
	Daily laborer	74	27.7	
Age at menarche (in years) (mean ± SD)		267	14.44 (± 1.08)	
Age at first intercourse (in years) (mean ± SD)		267	19.35 (± 1.82)	
Age at first marriage (in years) (mean ± SD)		217	22.51 (± 3.23)	
Age at first pregnancy (in years) (mean ± SD)		241	23.71 (± 3.23)	

*Adare, Somalia.

### Lifestyle and Behavioral Characteristics

Seven (2.6%) of the women in the study had a history of cigarette smoking, and only one woman reported being a current smoker. Fifty-two (19.5%) of study participants had a history of drinking alcohol, and 33 (12.4%) were current drinkers. One hundred seventy-four (65.1%) and 165 (75.7%) were multi-gravid and multi-para, respectively. Among the 232(86.9%) study participants who had a pregnancy history for more than 28 weeks, 57(24.6%) had one child, 107(46.1%) had two children, and 68(29.3%) had three or more children. Of 23 (9.5%) with a history of abortion, 14 (60.9%) participants were who had a history of abortion two or more times. Of the 214 (80.1%) modern family planning users, 125 (58.4%) used condoms and 56 (26.2%) used injectable family planning methods, respectively (**Table 2**).

**Table 2:** Lifestyle and behavioural characteristics of study participants in Saint Peter Specialized Hospital, ART clinic, Ethiopia, 2020 (n=267).

Variables	Categories	Frequency	Percentage (%)
Smoking history	Yes	7	2.6
	No	260	97.4
Current smoker	Yes	1	0.4
	No	266	99.6
Alcohol drinking history	Yes	52	19.5
	No	215	80.5
Current alcohol drinking	Yes	33	12.4
	No	234	87.6
No. sexual partner ever had	One	30	11.2
	≥ Two	237	88.8
Current sexual partner	None	73	27.3
	One	182	68.2
	≥ Two	12	4.5
Gravidity	Null gravid	26	9.7
	Gravida-1	67	25.1
	Gravida-2	102	38.2
	≥ Gravida-3	72	27
Parity	Para-1	53	24.3
	Para-2	97	44.5
	≥ Para-3	68	31.2
Number of children	One	57	24.6
	Two	107	46.1
	Three or more	68	29.3
Ever had abortion	Yes	23	9.5
	No	218	90.5
Number of abortion	Once	9	39.1
	Twice or above	14	60.9
Family planning use	Yes	214	80.1
	No	53	19.9
Type of family planning	Condom	125	58.4
	Pills	15	7.0
	Injectable	56	26.2
	Implant	10	4.7
	IUCD	7	3.3
	Permanent	1	0.5

### Reproductive and Other Clinical Health

Forty-seven (17.6%) of the participants had a history of STIs, and 18 (6.7%) had a family history of cervical cancer. One hundred thirteen (42.3%) of study participants had HIV for more than ten years, followed by 84 (31.5%) for six to ten years and 70 (26.2%) for one to five years. Participants in the study who had been on ART for 1-5, 6-10, and >10 years were 80 (30.0%), 94 (35.2%), and 93 (34.8%), respectively. Currently, 239 (89.5%) had good ART drug adherence, and 26 (9.7%) were placed on second-line ART drug regimens. At the outset, 104 (39.0%) and 22 (8.2%) of study participants were in WHO clinical stages III and IV, respectively. A baseline CD4 count of 200 cells/ml was found in 111 (41.6%) of the participants. The most recent viral load count of 230 (86.1%) study participants was undetectable. Vaginal good abnormality was found in 16 (6.0%) of the participants (**Table 3**).

**Table 3:** Reproductive health and clinical characteristics of participants in Saint Peter Specialized Hospital, ART clinic, Ethiopia, 2020 (n=267).

Variables		Frequency	Percentage (%)
History of STI	Yes	47	17.6
	No	220	82.4
Family history of cervical cancer	Yes	18	6.7
	No	249	93.3
Duration of HIV infection	1-5 years	70	26.2
	6-10 years	84	31.5
	>10 years	113	42.3
Duration on ART	1-5 years	80	30.0
	6-10 years	94	35.2
	>10 years	93	34.8
ART drug adherence	Good	239	89.5
	Fair	24	9.0
	Poor	4	1.5
Current ART drug regimen	1^st^ line	241	90.3
	2^nd^ line	26	9.7
Baseline clinical stage	I	90	33.7
	II	51	19.1
	III	104	39.0
	IV	22	8.2
Current clinical stage	I	264	98.9
	III	2	0.7
	IV	1	0.4
Baseline CD4 count (cell/ml)	<200	111	41.6
	200-350	81	30.3
	>350	75	28.1
Current CD4 count (cell/ml)	<200	35	13.1
	200-350	46	17.2
	>350	186	69.7
Viral load (copy/ml)	<1000	230	86.1
	≥1000	37	13.9
INH prophylaxis	Not initiated	88	33.0
	On INH	8	3.0
	Completed	157	58.8
	Defaulted	14	5.2
Previous TB infection	yes	61	22.8
	No	206	77.2
Vaginal good abnormality	Yes	16	6.0
	No	251	94.0

### Prevalence of Precancerous Cervical Lesion

The prevalence of precancerous cervical lesions among ART clients in St. Peter Specialized Hospital was 20(7.5%) with 95%CI of (4.1%-10.5%).

### Factors Associated With Precancerous Cervical Lesion

In bivariate binary logistic regression analysis, age, age at first sexual intercourse, age at first pregnancy, modern family planning use, previous STI, duration on ART, current ART drug regimen, drug adherence, and viral load of mothers were significantly associated with the precancerous cervical lesion. But, in the multivariable binary logistic regression analysis, modern family planning use, previous STI, and viral load were factors significantly associated with a precancerous cervical lesion (**Table 4**).

**Table 4:** Bivariate analysis of sociodemographic, reproductive and clinical health with precancerous cervical lesion in SPSH ART clinic, 2020.

Variables		VIA^+^	VIA^-^	COR (95%CI)	p-value
Age	≤40	8	155	1	
	>40	12	92	2.527 (0.996-6.412)	0.051
Age at first sexual intercourse	≤20	10	192	0.286 (0.113-0.723)	0.008
	>20	10	55	1	
Age at first pregnancy	≤25	13	173	0.751 (0.256-2.209)	0.603
	>25	5	50		
Family planning	Yes	11	203	1	
	No	9	44	3.775 (1.476-9.657)	0.006
STI	Yes	8	39	3.556 (1.364-9.265)	0.009
	No	12	208	1	
Duration on ART in years	1-5	4	76	1	
	6-10	6	88	0.437 (0.132-1.451)	0.176
	>10	10	83	0.566 (0.197-1.626)	0.290
Current ART drug regimen	1^st^ line	14	227	1	
	2^nd^ line	6	20	4.864 (1.685-14.040)	0.003
ART drug adherence	Good	14	225	1	
	Poor	6	22	4.383 (1.531-12.548)	0.006
Viral load (copy/ml)	<1000	7	24	1	
	≥1000	13	223	17.256 (6.280-47.412)	0.000
No. of sexual partner ever had	One	3	27	1	
	≥Two	17	220	1.440 (0.396-5.236)	0.581
Number of current sexual partner	None	7	66	1	
	One	10	172	0.548 (0.200-1.500)	0.242
	≥Two	3	9	3.143 (0.686-14.388)	0.140
Alcohol drinking history	Yes	5	47	1.418 (0.491-4.097)	0.518
	No	15	200	1	
Current alcohol drinking	Yes	4	29	1.872 (0.588-6.008)	0.287
	No	16	218	1	

The odds of developing precancerous cervical lesion among women who did not use modern family planning was 4.14 times the odds of those women who used modern family planning (AOR = 4.14; 95% CI = 1.23-13.87). The odds of developing precancerous cervical lesion among women who had a history of STI was 5.39 times the odds of those women who had no history of STI (AOR=5.39, 95% CI =1.56-18.70). The odds of developing precancerous cervical lesion among women who had a high viral load (≥1000 copy/ml) was 20.85 times the odds of those women who had suppressed viral load (<1000copy/ml) (AOR=20.85, 95% CI= 6.19-70.25) (**Table 5**).

**Table 5:** Multivariable analysis of sociodemographic, reproductive, and clinical health with the precancerous cervical lesion in Saint Peter Specialized Hospital, ART clinic, Ethiopia, 2020.

Variables	Categories	Precancerous cervical lesion		AOR (956%CI)	P-value
		Visual Inspection with Acetic Acid Positive (VIA+)	Visual Inspection with Acetic Acid Negative (VIA-)		
Age in years	≤40	8	155	1	
	>40	12	92	1.90 (0.55-6.56)	0.310
Age at first sexual intercourse (in years)	≤20	10	192	0.95 (0.22-4.00)	0.940
	>20	10	55	1	
Age at first pregnancy (in years)	≤25	13	173	0.68 (0.18-2.61)	0.570
	>25	5	50	1	
Modern family planning	Yes	11	203	1	
	No	9	44	4.14 (1.23-13.87)*	**0.021**
History of STI	Yes	8	39	5.39 (1.56-18.70)*	**0.008**
	No	12	208	1	
Duration of ART in years	1-5	4	76	1	
	6-10	6	88	1.49 (0.27-8.33)	0.647
	>10	10	83	3.19 (0.70-14.52)	0.133
Current ART drug regimen	1^st^ line	14	227	1	
	2^nd^ line	6	20	0.45 (0.06-3.58)	0.447
ART drug adherence	Good	14	225	1	
	Poor	6	22	0.90 (0.12-6.68)	0.920
Viral load (copy/ml)	<1000	7	24	1	
	≥1000	13	223	20.85 (6.19-70.25)*	**0.001**
No. sexual partner ever had	One	3	27	1	
	≥Two	17	220	2.80 (0.07-11)	0.587
Number of current sexual partners	None	7	66	1	
	One	10	172	0.64 (0.15-2.64)	0.535
	≥Two	3	9	6.15 (0.48-78.11)	0.162
Alcohol drinking history	Yes	5	47	1.45 (0.13-16.07)	0.762
	No	15	200	1	
Current alcohol drinking	Yes	4	29	3.76 (0.81-17.52)	0.091
	No	16	218	1	

NB: * Significantly associated variables at p-value <0.005.

## Discussion

This study assessed the magnitude of the precancerous cervical lesion and its associated factors among adult women with HIV on Anti Retroviral Therapy at Saint Peter Specialized Hospital, Ethiopia. It revealed that the magnitude of the precancerous cervical lesion among adult women with HIV on Anti Retroviral Therapy was 7.5% (95%CI: 4.1%-10.5%). HIV-positive women who did not utilize modern family planning, who had a history of STI and poor viral suppression were significantly associated with precancerous cervical lesions.

According to a finding of this study the prevalence of precancerous cervical lesions among HIV-positive women attending ART clinics in St. Peter Specialized Hospital was 7.5%. A similar finding was reported in a study conducted in Nigeria (6%) (23) and Uganda (6.3%) (24). This could be due to the fact that they use a similar strategy and methods for screening and have a similar social structure. In contrast to this, the finding of this study is lower than the studies done in Zambia (52%) (25), Kenya (26.7%), (26), and Brazil (23.4%) (27). The observed inconsistency in the prevalence of precancerous cervical lesions could be attributed to differences in sociodemographic or clinical characteristics of study participants, such as age composition, ART initiation, and adherence to ART drugs. Differences in precancerous cervical lesion screening methods could also explain the observed discrepancy in precancerous cervical lesion prevalence.

In this study, the odds of developing precancerous cervical lesion among women who did not use modern family planning was 4.14 times the odds of those women who used modern family planning methods. This could be related to the fact that the majority of modern family planning methods thicken the cervical mucus, which helps to prevent the entry of infections such as HPV, which is a widely accepted cause of cervical cancer. In contrast to this, a finding from a few studies suggests that oral contraceptive use increased the occurrence of precancerous cervical lesions as prolonged use of oral contraceptives may increase the eversion of the columnar epithelium to the ectocervix, which enhances the exposure of the columnar epithelium to HPV infection (7, 28, 29). Our study pointed out that nearly 60% of the 80.1% of family planning users were condom users, which have a dual purpose of family planning and STI prevention, including human papillomavirus (HPV) infection. This could explain why, in our study, modern family planning had a protective effect on the precancerous cervical lesion. Condom use was consistently advised to HIV patients; this could be the most effective method of protecting against STIs, which were the most risk factor for Cervical Cancer.

This study pointed out that the odds of developing precancerous cervical lesion among women who had a history of STI was 5.39 times the odds of those women who had no history of STI. One possible explanation could be the co-infection of HPV with other STIs, which severely affects immunocompromised women and makes them vulnerable to other opportunistic infections. This is in harmony with studies done in Addis Ababa (30) and Adama (7) of Ethiopia which revealed that women with a history of STIs were significantly associated with the cervical precancerous lesion. Another study in Uganda found that women who did not have STI at the time of their visit had a 76% lower risk of developing precancerous cervical lesions (24). The majority of evidence suggested that cervical cancer is caused by a viral infection and that the virus spreads through sexual contact. Chlamydia trachomatis infection, for instant, has been linked to the severity of abnormal cervical cytology and HPV infection.

According to the findings of the current study, the odds of developing precancerous cervical lesion among women who had a high viral load (≥1000 copy/ml) was 20.85 times the odds of those women who had suppressed viral load <1000 copy/ml). Clients’ immune status is directly related to their CD4 count and HIV-viral load. A high viral load indicates immune suppression and a poor response to ART medications. Similarly, a follow-up study at the Chittaranjan National Cancer Institute found that women with high viral loads had a fourfold higher risk of cervical intraepithelial neoplasia than those with low viral loads (20). This could be because HPV DNA integration into host cells is required for malignant transformation by the E6 and E7 proteins. HPV DNA load has been proposed as a risk factor for dysplasia and cervical cancer.

### Limitation of the Study

Visual inspection with an acetic acid test result of postmenopausal women may not be reliable, because the transformation zone of these women is often inside the cervical canal which may have an impact on the magnitude.

## Conclusions

In this study, the prevalence of precancerous cervical lesions was relatively lower than what had been reported in low and middle-income countries. Modern family planning, history of sexual transmitted infection, and viral load had a significant association with a precancerous cervical lesion. Hence, encouraging modern family planning, and routine screening of women for pre-cancerous cervical lesions for those with high viral load have enormous contributions to decreasing cervical cancer disease among Women with Human Immune Deficiency Virus through Anti Retroviral Therapy.

## Data Availability Statement

The raw data supporting the conclusions of this article will be made available by the authors, without undue reservation.

## Ethics Statement

The studies involving human participants were reviewed and approved by Haramaya University College of Health and Medical Sciences Institutional Health Research Ethics Review Committee (IHRERC). The patients/participants provided their written informed consent to participate in this study.

## Author Contributions

All authors made a significant contribution to the work reported, whether that is in the conception, study design, execution, acquisition of data, analysis, and interpretation, or in all these areas; took part in drafting, revising, or critically reviewing the manuscript; gave final approval of the version to be published; have agreed on the journal to which the article has been submitted; and agree to be accountable for all aspects of the work.

## Funding

Haramaya University provided financial support for this study. But the funding agency had no role in the collection, analysis, and interpretation of the data as well as the writing-up of the manuscript.

## Conflict of Interest

The authors declare that the research was conducted in the absence of any commercial or financial relationships that could be construed as a potential conflict of interest.

## Publisher’s Note

All claims expressed in this article are solely those of the authors and do not necessarily represent those of their affiliated organizations, or those of the publisher, the editors and the reviewers. Any product that may be evaluated in this article, or claim that may be made by its manufacturer, is not guaranteed or endorsed by the publisher.

## Abbreviations

CC, Cervical Cancer; CI, Continuous Integration; CIN, Cervical Intraepithelial Neoplasia; HSV, Herpes Simplex Virus; ICC, Invasive Cervical Cancer; PCL, Precancerous Cervical Lesions; PLHIV, People Living With Human Immunodeficiency Virus; OC, Oral Contraceptive; SPSH, Saint Peter Specialized Hospital.
