# Supplementary material for: Precancerous Cervical Lesion Among Adult Women With Human Immune Deficiency Virus on Anti Retroviral Therapy At Saint Peter Specialized Hospital, Ethiopia: A Hospital-Based Cross-Sectional Study
Source: Front Oncol. 2022 Jul 22;12:910915. doi: 10.3389/fonc.2022.910915 (PMC9361014; doi:10.3389/fonc.2022.910915)
Supplement: Supplementary file 1 [file DataSheet_1.pdf]

7.3. Questionnaire (English version)

Questioner on assessment of prevalence precarious of cervical lesion and associated factors among adult women with HIV on ART at Saint Peter Specialized Hospital ,Addis Ababa.

Identification Information.001. Code No. -----------------

Date of interview ___/___/___

Instruction for interviewer: For each of the following questions, please circle the number of the alternative or fill the blank space that fit the response of the respondent

Part I: Socio-demographic characteristics

Ser.No	Variables	Categories	Skip
101	Age in years	________ years	
102	Residence	1,Urban<br>2,Rural	
103	Religion	1,Orthodox<br>2,Muslim<br>3,Protestant<br>4,Catholic<br>5,Others __________(specify)	
104	Ethnicity	1,Amahara<br>2,Oromo	

		3,Tigray<br><br>4,Gurage<br><br>5,Others_________(specify)	
105	Educational status	1,Illiterate<br><br>2,Able to read and write<br><br>3,Primary school (1-8th grade)<br><br>4,Secondary school (9th - 12th grade)<br><br>5,College and above	
106	Marital status	1,Single<br><br>2,Married<br><br>3,Separated<br><br>4,Widowed<br><br>5,Divorced<br><br>6,Others _________(specify)	
107	Occupation	1,House wife<br><br>2,Merchant<br><br>3,Government employ<br><br>4,Private employee<br><br>5,Daily laborer	

		6,Farmer<br><br>7,Others ________(specify)	

Part II: Socio-economic status (wealth index assessment tool)

No.	Questions	Alternative	Skip
201.	What kind of house hold materials do you have?	1,Electricity<br>2,Telephone<br>3,Television<br>4,Refrigerator<br>5,Bed<br>6,Sofa<br>7,Radio<br>8,Bicycle<br>9,Motorcycle<br>10,Car<br>11,Others (____________ specify)	
202.	Source of drinking water	1,Pipe in residence<br>2,Pipe in public tap<br>3,Well in residence<br>4,Public well<br>5,Surface water<br>6,Spring water<br>7,River<br>8,Others (____________ specify)	
203.	Do you have a latrine?	1,Yes<br>2,No	If No, skip to Q205
204.	If yes to Q203, what type of latrine?	1,Own flush toilet<br>2,Shared flush toilet<br>3,Pit latrine<br>4,Ventilated pit latrine<br>5,Open field<br>6,Others (____________ specify)	
205.	What is the floor material (for the main house)?	1,Earth<br>2,Cow dung<br>3,Cement<br>4,Wooden<br>5,Other (____________ specify)	
206.	What is the roofing material (for the main house)?	1,Corrugated iron sheet<br>2,Thatch or grass<br>3,Wood and mud<br>4,Mud and stone<br>5,Reed and bamboo	

		6,Others (____________ specify)	
207.	What is the wall material (for the main house)?	1,Wood and mud<br>2,Reed and bamboo<br>3,Stone and mud<br>4,Stone & cement<br>5,Bricks<br>6,Others (____________ specify)	
208.	Number of member per sleeping room	____________	
209.	Dose the household has a domestic worker not related to head	1,Yes<br>2,No	
210.	Separate house for animals	1,Yes<br>2,No	
211.	Do you have a house?	1,Yes<br>2,No	If No, skip to Q213
212.	If yes to Q211, is it	1,Private<br>2,Rent	
213.	Are there any windows in the house?	1,Yes<br>2,No	
214.	What do you use for lighting	1,Electricity<br>2,Gas/kerosene<br>3.Solar<br>4,Others (____________ specify)	
215.	What do you use for cooking	1,Wood<br>2,Dung<br>3,Charcoal<br>4,Gas<br>5,Biogas<br>6,Electricity<br>7,Others (____________ specify)	

Part iii Behavoural related factors

S.No	Variables	Categories	Skip
301	Have you ever smoke cigarette?	1,yes<br><br>2,No	
302	Do you currently smoke cigarette?	1,yes<br><br>2,No	If No, skip to Q305
303	If yes to Q302, how many cigarette per day?	________ /day<br><br>________/week	
304	If yes to Q302, how long since you started smoking?	_________ years	
305	Have you ever drink alcohol?	1,yes<br><br>2,No	
306	Do you currently drink alcohol?	1,yes<br><br>2,No	If No, skip to Q309
307	If yes to Q306, how often do you drink?	1,Always<br><br>2,Sometimes<br><br>3,Rarely	
308	If yes to Q306, how long since you started drinking alcohol	_____years alcohol	

309	How many sexual partners have you ever had in your	____life time?	
310	Current number of sexual partner?	_______________	

Part IV: Sexual and reproductive health related questions

S.No.	Variables	Categories	Skip
401	Age at menarche	_______ years<br>I don't remember	
402	Age at first sexual intercourse	_______ years<br>I don't remember	
403	Age at first marriage (for those who are not single)	_______ years<br>I don't remember	
404	Have you ever been pregnant?	1,Yes<br>2,No	If No, skip to Q411
405	Age at first pregnancy	_______ years<br>I don't remember	
406	Gravidity	____________	
407	Parity	____________	

408	How many children do you have?	__________	
409	Did you have history of abortion?	1,Yes<br><br>2,No	If No, skip to Q411
410	If yes, how many abortions did you experienced?	_________	
411	Type of abortion?	Spontaneous<br><br>Induced<br><br>Others (_________specify)	
412	If induced, where did you get abortion service	1,Health facility<br><br>2,Traditional healer<br><br>3,Others	
413	Have you ever used any of modern family planning methods?	1,Yes<br><br>2,No	If No, skip to Q413
414	If yes, which type of modern family planning methods did you use	1,Condom<br><br>2,Pills<br><br>3,Injectable<br><br>4,Implant	

		5,IUCD<br><br>6,Tubal ligation	
415	Have you ever experienced with abnormal vaginal discharge or ulceration around the genital area?	1,yes<br><br>2,No	
416	Have ever diagnosed with STI?	1,YES<br><br>2,NO	
417	Do you have family history of cervical cancer	1,YES<br><br>2,NO	
418	How long since you know you are infected with HIV? (in years)	________years	

Part V: HIV infection related questions

S.N.	Variables	Categories	Skip
501.	Date of ART initiation	___/_____/____________	
502.	ART drug regimen at the time of ART initiation	_____________	
503.	Current ART drug regimen	_____________	
504.	ART drug adherence status	1,Good<br>2,Fair<br>3,Poor	
505.	WHO clinical stage at the time of ART initiation	_________	
506.	Current WHO clinical stage	_________	
507.	CD4 count at the time of ART initiation	_________cell/ml	
508.	Current CD4 count	_________cell/ml	

509.	Latest viral load count	________copies/ml<br>Undetectable	
510.	Ever diagnosed with any form of TB	1,Yes<br>2,No	
511.	INH status	1,Not initiated<br>2,Completed<br>3,Defaulted/stopped<br>4,Others (_________specify)	
512.	Vaginal wall abnormality	1,Yes<br>2,No	
513	VIA test result	1,Negative<br>2,Positive<br>3,Suspicious	
